# Insight into Illness Among Inpatients in a National Forensic Mental Health Service: A Dundrum Forensic Redevelopment Evaluation Study (D-FOREST)

**DOI:** 10.1192/j.eurpsy.2024.344

**Published:** 2024-08-27

**Authors:** M. U. Waqar, S. Murray, A. O’Reilly, H. G. Kennedy, M. Davoren

**Affiliations:** ^1^Central Mental Hospital, National Forensic Mental Health Service, Portrane; ^2^Dundrum Centre for Forensic Excellence, Trinity College Dublin, Dublin, Ireland

## Abstract

**Introduction:**

Forensic psychiatric services serve a dual purpose: treatment of mental disorders and prevention of associated violent reoffending. Progression along the secure care pathway is often impeded by impaired insight, mainly as a result of treatment-resistant psychoses.

**Objectives:**

We assessed levels of insight among patients in Ireland’s National Forensic Mental Health Service before and after its relocation from the historic 1850 campus in Dundrum to a modern facility in Portrane, Dublin.

**Methods:**

The VAGUS insight scale was used in this repeated measures study before and after the relocation at two time points 42 months apart. All inpatients were invited to participate in completing the self-report (VAGUS-SR) and clinician-rated (VAGUS-CR) versions on both occasions. Total scores of both versions were averaged to obtain a combined VAGUS insight score. Corresponding Positive and Negative Syndrome Scale (PANSS) scores were used to ascertain correlations between the insight and symptomatology scales. This study is part of the Dundrum Forensic Redevelopment Evaluation Study (D-FOREST)

**Results:**

40 pairs of observations were available for legal capacity to consent to medication, combined VAGUS-CR and VAGUS-SR assessments of insight (Cronbach’s alpha=0.927), and PANSS. VAGUS-CR insight and PANSS scores were progressively better from admission and high dependency wards through medium-term medium secure wards to rehabilitation and pre-discharge wards. Mean scores did not change significantly over this time interval. Those legally certified fit to give or withhold consent by their treating consultant psychiatrists scored significantly better on the VAGUS combined insight scale: 8.3 (SD 1.7) v 5.3 (2.2) at baseline, paired *t*=25.9, *p*<0.001; and also 42 months later: 8.2 (1.4) v 5.7 (3.9), paired *t*=5.2, *p*=0.022. PANSS subscales were all significantly better for those assessed as being capacitous. Change in combined VAGUS score correlated with change in all PANSS subscales. Binary logistic regression with legal capacity as the dependent variable yielded a model in which combined VAGUS score and PANSS positive symptom score were independent determinants of assessed capacity status. Receiver operating characteristic area under the curve was 0.873, 95% CI 0.760-0.986, at baseline and 0.856, 95% CI 0.720-0.991, at 42 months. A score of 7.3 yielded a sensitivity of 0.8 and a specificity of 0.8.

**Conclusions:**

The combined VAGUS score is a reliable and valid measure of insight relevant to functional mental capacity to consent to treatment with sensitivity and specificity sufficient to guide but not bind clinical decision-making. It measures a quality that varies with symptom severity but is also partly independent of symptom severity; the constructive inclusion of self-reported insight is notable.

**Disclosure of Interest:**

None Declared

